# 

**Published:** 1899-11

**Authors:** 


### EDITORIAL.

### The Thirty-fourth Annual Meeting of the Ohio State Dental Society.

The thirty-fourth annual meeting of the Ohio State Dental Society will be held at the Great Southern Hotel in the city of Columbus on Tuesday, Wednesday and Thursday, December 5, 6 and 7, 1899.

A very interesting and, we trust, profitable program has been prepared which consists of essays and clinics. The essayists are among the most eminent in our profession.

Clinics will form an important part in this meeting. Many young men are among the clinicians, and we trust the profession will turn out and give them a hearty welcome and co-operation in their endeavors to assist in the success of our meeting.

The progressive dentist can not, in these closing years of this wonderful nineteenth century, remain at home and expect to gain from the journal reports all that takes place at the meeting. The fact of the matter is, that much of the good to be derived from attendance on our annual meetings is gained in the little side talks and conversations which naturally take place at such a time. Then, too, one meets many of those whom he knows only by name and is thus enabled to enlarge the scope of his acquaintance. Another feature of association is that one is stimulated, no matter how efficient he may be in his profession, to more perfect work and achievement.

Let this meeting be an enthusiastic one in point of numbers, at least, and we are sure you will go home refreshed and determined to achieve greater success in our beloved profession.

PROGRAM.

TUESDAY, DECEMBER 5TH—10 A. M.

ORDER OF BUSINESS.

Roll Call.

Reading of Minutes.

Reports of Committees.

Miscellaneous Business.

Reading of Papers and Discussions.

ESSAYS.

President's Address, L. P. Bethel, Kent.

TUESDAY—2 P. M.

G. S. Junkerman, Cincinnati—A Hydro-Mechanical Theory of Sensitive Dentin.

Discussion opened by F. S. Maxwell, Steubenville; C. T. Whinery, Toledo.

Interesting cases in practice: J. K. Douglas, Sandusky—Third Superior Molar in Antrum of Highmore.

H. C. Matlack, Cincinnati—Antral Disease in a Child.

L. E. Custer, Dayton—(Subject to be announced).

G. D. Edgar, Defiance—Inverted Third Molar.

GENERAL DISCUSSION.

T. A. Long, Philadelphia, Pa.—Japanese Dental Art, Ancient and Modern. Illustrated by specimens.

GENERAL DISCUSSION.

5 P. M.—Election of Officers.

TUESDAY—7:30 P. M.

W. A. Price, Cleveland—Lecture and Demonstration of Roentgen Rays as applied to Dentistry. (Also lantern views).

Discussion opened by L. E. Custer, Dayton; J. S. Cassiday, Covington, Ky.

WEDNESDAY, DECEMBER 6TH—9 A. M.

CLINICS—OPERATIVE.

1. E. D. Scheble, Toledo—Gold Filling by Hand Pressure.
2. C. I. Keely, Hamilton—Tin and Gold Filling.
3. J. R. Owens, Cleveland—Contour Filling with Woodward Matrix.
4. G. W. Woodbury, Cleveland—Immediate Pulp Removal.
5. D. H. Sullivan, Lima—The Preparation of Sensitive Teeth for Filling, by the use of Nitrous Oxide and Chloroform.
6. W. H. Hayden, Youngstown—Electric Water Heater and Gold Plating.

SURGICAL.

7. J. R. Bell, Cleveland—New Instruments for Removing Soft Tissue from Partially Erupted Teeth.

PROSTHODONTIA.

8. W. T. Jackman, Cleveland—Putting on and Baking of Body and Enamel Continuous Gum Plate. (Using electric furnace).

9. H. A. Moyer, Kendallville, Ind.—Something Novel in Shot Swaging.

10. W. O. Hulick, Cincinnati—Crown and Bridge-work.

11. S. M. Weaver, Cleveland—Novelties in Crown and Bridge-work.

ORTHODONTIA

12. V. E. Barnes, Cleveland—Impressions and Casts for use in Orthodontia.

METALLURGY.

13. A. A. Kumler, Cincinnati—Refining and Rolling Gold.

WEDNESDAY—2 P. M.

H. J. Custer, Columbus—Reflexes between the Teeth and Eye and Ear.

Discussion opened by Grant Molyneaux, Cincinnati; O. E. McConkey, Urbana.

M. H. Fletcher, Cincinnati—Alkaline Saliva.

Discussion opened by J. R. Callahan, Cincinnati; S. B. Dewey, Cleveland.

H. F. Harvey, Cleveland—A New Form of Clamp.

Voluntary Essays.

WEDNESDAY EVENING—OPEN.

THURSDAY, DECEMBER 7TH—9 A. M.

Installation of Officers.

CLINICS—9:30 A. M.—OPERATIVE.

1. L. L. Barber, Toledo—Gold and Platinum Filling with Electric Mallet.

2. C. R. Butler, Cleveland—Gold Lined Tin Filling.

3. W. T. McLean, Cincinnati—Difficult Filling by use of Automatic Plugger.

4. Otto Arnold, Columbus—Pressure Anesthesia for Immediate Pulp Removal.

5. Hamlin Barnes, Wellsville—Dr. Fell's Method of Forced Respiration.

6. Ira Brown, Cleveland—Phagedenic Pericementitis.

7. David Stern, Cincinnati—Devitalizing and Bleaching with Electric Current.

SURGICAL.

8. H. C. Matlack, Cincinnati—Opening Antrum with Surgical Engine.

PROSTHODONTIA.

9. W. T. Born, Kenton—Attachment of Clasps for Plate Dentures.

10. J. B. Snyder, Bryan—Impressions.

11. S. D. Ruggles, Portsmouth—The use of Pure Gold Wire in Crown-work.

12. J. F. Stephan, Cleveland—Preparing Roots and Fitting Bands for Crowns.

13. L. H. McDonald, Norwalk—One way of Fastening Brdges.

ORTHODONTIA.

14. W. S. Locke, Cincinnati—The Making of Regulating Appliances.

METALLURGY.

15. E. B. Lodge, Cleveland—Repairing and Tempering Instruments from old Excavators.

